# Hemorrhage Caused by Snake Venom Metalloproteinases: A Journey of Discovery and Understanding †

**DOI:** 10.3390/toxins8040093

**Published:** 2016-03-26

**Authors:** José María Gutiérrez, Teresa Escalante, Alexandra Rucavado, Cristina Herrera

**Affiliations:** 1 Instituto Clodomiro Picado, Facultad de Microbiología, Universidad de Costa Rica, San José 11501-2060, Costa Rica; teresa.escalante@ucr.ac.cr (T.E.); alexandra.rucavado@ucr.ac.cr (A.R.); cristina.herreraarias@gmail.com (C.H.); 2 Facultad de Farmacia, Universidad de Costa Rica, San José 11501-2060, Costa Rica

**Keywords:** snake venom, viperids, metalloproteinases, hemorrhage, capillary vessels, basement membrane, type IV collagen

## Abstract

The historical development of discoveries and conceptual frames for understanding the hemorrhagic activity induced by viperid snake venoms and by hemorrhagic metalloproteinases (SVMPs) present in these venoms is reviewed. Histological and ultrastructural tools allowed the identification of the capillary network as the main site of action of SVMPs. After years of debate, biochemical developments demonstrated that all hemorrhagic toxins in viperid venoms are zinc-dependent metalloproteinases. Hemorrhagic SVMPs act by initially hydrolyzing key substrates at the basement membrane (BM) of capillaries. This degradation results in the weakening of the mechanical stability of the capillary wall, which becomes distended owing of the action of the hemodynamic biophysical forces operating in the circulation. As a consequence, the capillary wall is disrupted and extravasation occurs. SVMPs do not induce rapid toxicity to endothelial cells, and the pathological effects described in these cells *in vivo* result from the mechanical action of these hemodynamic forces. Experimental evidence suggests that degradation of type IV collagen, and perhaps also perlecan, is the key event in the onset of microvessel damage. It is necessary to study this phenomenon from a holistic, systemic perspective in which the action of other venom components is also taken into consideration.

## 1. Introduction

Snakebite envenoming constitutes a highly relevant, albeit largely neglected, public health problem on a global basis, affecting primarily impoverished populations in the rural settings of Africa, Asia, Latin America and parts of Oceania [1,2,3]. Although accurate statistics on incidence and mortality due to snakebite envenoming are scarce, it has been estimated that between 1.2 and 5.5 million people suffer snakebite envenomings every year, with 25,000 to 125,000 deaths, and an estimated number of 400,000 victims left with permanent sequelae [4,5].

The clinical manifestations of envenomings vary depending on the species of snake causing the bite, and there is a large spectrum of pathophysiological effects induced by snake venoms, owing to the diverse arsenal described in their composition [6]. In addition, other factors play a key role in the severity of envenomings, such as the volume of venom injected, the anatomical site of the bite, and the physiological characteristics of the affected person [2]. Envenomings by snakes of the family Viperidae, and by some species of the family “Colubridae” (*sensu lato*), are characterized by prominent local and systemic hemorrhage. Blood vessel damage leading to extravasation, in turn, contributes to local tissue damage and poor muscle regeneration, and to massive systemic blood loss leading to hemodynamic disturbances and cardiovascular shock [2,7].

The study of the mechanism by which snake venoms induce hemorrhage and the characterization of hemorrhagic toxins and their mechanism of action has been a fascinating area of research within the field of Toxinology. The present contribution summarizes the journey of discovery and understanding that has led to our current view of snake venom-induced hemorrhage, and highlights some of the seminal discoveries and hypotheses generated during more than a century of scientific efforts.

## 2. Describing the Occurrence of Hemorrhage in Clinical and Experimental Viperid Snakebite Envenomings

Hemorrhage was recognized as a frequent and relevant manifestation early on in the description of the main clinical features of viperid snakebite envenomings, and bleeding in various organs was identified as one of the most serious consequences of these envenomings (see for example [8,9,10]). At the experimental level, J.B. de Lacerda, in 1884 [11], working in Brazil, described the effects induced by *Bothrops* sp. venoms in experimental animals. In his recount on local pathological effects, he reported that “*Le tissu cellulaire sous-cutané*, *est tout infiltré et présente de place en place des taches noiràtres*, *des points violacés et épars*, *des nuances livides diffuses et de nombreuses extravasions sanguines… Le tissu cellulaire*, *qui cuvre les muscles*, *est également infiltré*, *il offre un aspect gélatineux et est plus ou moins imbibé du sang noir*, *en partie coagulé*”. Two years later, Mitchel and Reichert, in the USA, described hemorrhagic events in animals treated with viperid snake venoms [12]. When reporting an experiment performed in a rabbit, the authors stated that “*On the peritoneum were placed a few small particles of the dried venom of* Crotalus adamanteus. *In two or three minutes some extravasations appeared immediately about the point of the application of the venom*; *a few moments later these extravasations were diffused over a considerable area and had run into each other to such an extent as to form a patch of bleeding surface*”.

Other scientists investigated the hemorrhagic activity of viperid snake venoms in the first half of the 20th century by describing macroscopic observations in affected tissues, and by introducing the histological analysis of hemorrhagic lesions in organs. Likewise, researchers demonstrated the action of viperid venoms on the coagulation system and on platelets, and speculated on the contribution of these effects in the pathogenesis of venom-induced hemorrhage (reviewed in [13]). Thus hemorrhagic activity of snake venoms was a topic of interest for early clinical and experimental toxinologists owing to the relevance of hemorrhagic manifestations in the overall pathophysiology of viperid snakebite envenoming.

## 3. Devising Methods to Quantify Venom-Induced Hemorrhage

The experimental study of venom-induced hemorrhage and the identification of hemorrhagic components in snake venoms demanded the development of simple methods to quantify the extent of hemorrhagic lesions. One of the most significant advances towards this goal was achieved by the group of Akira Ohsaka in Japan [14]. A method was developed, consisting in the intracutaneous injection of various amounts of venom of *Trimeresurus* (now *Protobothrops*) *flavoviridis* into the depilated back skin of rabbits, followed by the measurement, 24 h later, of the size of the hemorrhagic spots in the inner side of the skin. The log dose–response curves showed that linear responses were obtained within a range of diameters from 10 mm to 18 mm. This skin method was then adapted for use in rats [15] and mice [16,17,18], with variations in the time lapse between venom injection and observation of hemorrhagic lesions. The hemorrhagic activity of a venom or toxin is expressed as the Minimum Hemorrhagic Dose (MHD), usually defined as the dose of venom or toxin that induces a hemorrhagic spot of 10 mm diameter [15,18]. This method has the limitation that it does not take into consideration the intensity of the hemorrhagic area, only its size. This has been circumvented by the quantification of the hemoglobin present in the hemorrhagic area of the skin [19].

Other methods for quantification of venom-induced hemorrhagic activity have been described [20], but modifications of the skin-based method of Kondo *et al.* [14] have been predominantly used in toxinological research owing to its simplicity and quantitative nature. It has been particularly useful in the assessment of hemorrhagic activity during fractionation of snake venoms for the purification of hemorrhagic components. On the other hand, methods have been established for the quantification of hemorrhagic activity by snake venoms in organs. The simplest one is based on the intramuscular or intravenous injection of venoms in mice followed by the euthanasia of animals and the sampling of affected organs. Tissues are then homogenized and the amount of hemoglobin in the tissue is quantified spectrophotometrically, by recording the absorbance at 540 nm [18,21]. For the quantification of hemorrhagic activity of venoms in the lungs, in addition to the method of Bonta *et al.* [20], the estimation of the Minimum Pulmonary Hemorrhagic Dose (MPHD) has been used. For this, groups of mice are injected intravenously with various doses of venom or toxin. After one hour, animals are euthanized and the thoracic cavity opened for observation of hemorrhagic spots in the surface of the lungs. The MPHD corresponds to the lowest venom dose which induces hemorrhagic lesions in the lungs in all mice injected [22].

## 4. Characterizing the Biochemical Properties of Hemorrhagic Toxins Present in Snake Venoms

Flexner and Noguchi [23] used the term “haemorrhagin” to denote the principle in snake venom responsible for hemorrhagic activity, and described it as “*the chief toxic constituent in Crotalus venom*”. By the middle of the 20th century, the hemorrhagic activity of viperid venoms was attributed by several researchers to the proteolytic activity of venoms [24], although the evidence for this hypothesis was mainly the fact that venoms with hemorrhagic activity also had high proteolytic action (see [13]). In addition, the inhibition of hemorrhagic activity by incubation with the chelating agent EDTA underscored the role of proteolytic activity in this effect [25]. However, it was not until modern chromatographic procedures were introduced in the study of snake venoms that this issue was properly addressed.

The early attempts to purify hemorrhagic toxins from snake venoms, by using electrophoretic and chromatographic methods, resulted in the isolation of proteinases devoid of hemorrhagic activity and of hemorrhagic toxins either having or lacking proteinase activity [26,27,28]. Thus, it was not completely clear at that time whether all viperid hemorrhagic toxins were proteinases or not. A source of confusion in these studies was the selection of the substrates for testing proteinase activity, as many of them followed the method of Kunitz [29], which uses casein as substrate, and assesses proteolytic activity by detecting acid-soluble peptides after precipitation of the enzyme-substrate mixture with trichloroacetic acid. Owing to the substrate specificity of snake venom hemorrhagic principles, this method is unable to detect proteolytic activity in some cases [30].

The advent of modern chromatographic procedures, especially after the decade of 1960, paved the way for the isolation of hemorrhagic components of good purity from a variety of viperid snake venoms. Among others, significant contributions in the purification of these components were performed by A. Ohsaka and colleagues in Japan [13], Grotto *et al.* in Israel [27], and F.R. Mandelbaum and coworkers in Brazil [31]. In 1978, Jon B. Bjarnason and Anthony T. Tu published a landmark study on the characterization of five hemorrhagic toxins from the venom of the rattlesnake *Crotalus atrox* [17]. Instead of employing the Kunitz’s casein method for assaying proteolytic activity, they used dimethylcasein as substrate, which has a higher sensitivity. They demonstrated that these hemorrhagic toxins were metalloproteinases containing one mol of zinc per mol of toxin. Chelating agents eliminated both proteinase and hemorrhagic activities and, when zinc was removed, the toxins lost both actions to a similar degree. As there was not a correlation between the extent of hemorrhagic and proteinase activities, the authors suggested that these toxins have a highly selective substrate specificity for inducing hemorrhage. This study clarified a number of apparent contradictions in the characterization of snake venom hemorrhagic components, and paved the way for a new age in the biochemical characterization of hemorrhagic toxins.

Thereafter, many hemorrhagic proteinases, currently known as “snake venom metalloproteinases” (SVMPs), have been purified and characterized from snake venoms (see reviews [32,33]). All hemorrhagic SVMPs are zinc-dependent proteolytic enzymes that, together with the ADAMS (“*a d*isintegrin *a*nd *m*etalloproteinase”) belong to the M12B family of metalloproteinases which, in turn, are grouped within the Metzincin clan, characterized by a canonical sequence in the zinc-binding region at the catalytic site and by a Met-turn [34]. SVMPs have been classified in three classes (PI, PII, and PIII) on the basis of their domain composition. In the mature protein, PI SVMPs comprise the metalloproteinase domain only, whereas PII SVMPs present a disintegrin (Dis) domain in addition to the catalytic domain. On the other hand, PIII SVMPs present metalloproteinase, disintegrin-like (Dis-like), and cysteine-rich (Cys-rich) domains. In turn, several subclasses have been described within each class, depending on whether they are monomers or homo- or heterodimers, and also on the variable patterns of post-translational cleavage of several domains [35]. The variations in domain composition between different classes of SVMPs have implications in the mechanism of action of hemorrhagic toxins (see Section 9 of this review). The detailed analysis of the biochemical characterization of SVMPs, as well their molecular evolution after the recruitment of an ADAM-like gene early on in the course of advanced snakes diversification are beyond the scope of this review; readers are referred to excellent publications on these topics [33,35,36].

## 5. Exploring the Pathological Effects Induced by Venoms and SVMPs on the Microvasculature

One of the first histological analyses of snake venom-induced hemorrhage was published by Taube and Essex [37]. It was observed that rattlesnake venom affected the integrity of endothelial cells in vessels, inducing them to swell, and then burst and dissolve, leaving gaps in the vessel walls. Moreover, the authors described loss in blood coagulability. Further histological studies described hemorrhage, *i.e.*, presence of erythrocytes in the interstitial space of various tissues, as a consequence of injection of viperid venoms [38]. These authors also described pathological changes in the arterial walls. However, the characterization of the precise morphological alterations induced by venoms in the structure of microvessels had to wait for ultrastructural studies using the transmission electron microscope (TEM). Early TEM observations in tissues injected with a purified hemorrhagic toxin and the venom of *T. flavoviridis* revealed erythrocytes escaping through the intercellular junctions at the endothelial cell lining [39]. It was proposed that the basement membrane (BM) adjacent to the intercellular junctions has to be disrupted to allow the extravasation [13]. Moreover, by observing the mesentery of rats using cinematographic techniques at the microscope, Ohsaka *et al.* [40] described an initial vasoconstriction of arterioles, followed by vasodilation and then by the extravasation of erythrocytes one by one through holes formed in the capillaries, but without an overt rupture of the endothelium. Taken together, these findings suggested that hemorrhage occurs by extravasation of erythrocytes through openings in the intercellular endothelial cell junctions. As to the mechanisms of this opening, it was suggested that inflammatory mediators released by hemorrhagic toxins, such as histamine, serotonin and others, are responsible for this phenomenon [13].

A different picture emerged with a detailed characterization of ultrastructural alterations in capillary vessels after a subcutaneous injection of a hemorrhagic toxin from the venom of *Vipera palestinae* (currently *Daboia palestinae*) [41]. Ultrastructural lesions were described in capillary endothelial cells, and erythrocytes escaped through damaged endothelial cells and not through widened intercellular junctions. Similar and more detailed ultrastructural observations were carried out in muscle tissue injected with hemorrhagic SVMPs and crude venom of *Crotalus atrox* [42,43]. When examining the morphology of affected capillaries, endothelial cells showed dilatation of endoplasmic reticulum, formation of blebs, intracellular swelling, drop in the number of pinocytotic vesicles and, most importantly, disruption and formation of gaps within the cells through which erythrocytes escaped. The basal lamina was damaged in some portions and, interestingly, intercellular junctions between endothelial cells were not affected. Ownby *et al.* [42,43] named this mechanism “*hemorrhage per rhexis*”, in contrast with the mechanism proposed by Ohsaka and colleagues, which was named “*hemorrhage per diapedesis*”.

**Figure 1:**
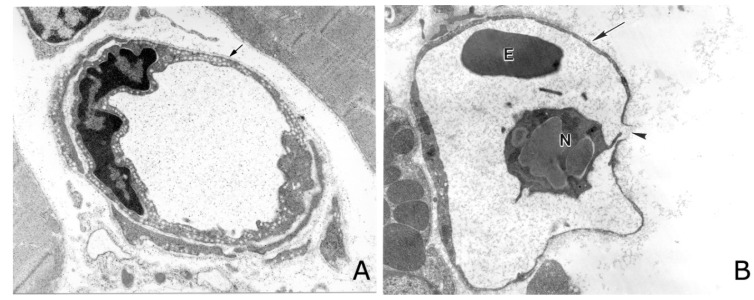
Pathological effects induced by a hemorrhagic snake venom metalloproteinase (SVMP) in capillary vessels. (**A**) Electron micrograph of a capillary vessel from muscle tissue injected with saline solution. Normal ultrastructure is observed in endothelial cell, including the presence of pynocytotic vesicles, and basement membrane (arrow). (**B**) Micrograph of a section of tissue injected with the hemorrhagic SVMP BaP1, from the venom of *Bothrops asper*. Notice prominent damage of endothelial cell, with loss of pynocytotic vesicles, distention and thinning of the cell, and rupture of cell integrity at one point (arrowhead). The basement membrane is absent along most of the periphery of the capillary (arrow). An erythrocyte (E) and a neutrophil (N) are observed inside the vessel. Magnification: 17,000× (**A**); and 10,000× (**B**). Reproduced by [47], Copyright 2006, Elsevier.

Afterwards, a number of groups investigated the morphological alterations in the microvasculature associated with the action of hemorrhagic SVMPs [22,44,45,46,47]. All of them described the extravasation of erythrocytes through lesions in capillary endothelial cells, and not through widened intercellular junctions, thus supporting the mechanism of hemorrhage *per rhexis*. (Figure 1).

In contrast, Gonçalves and Mariano [48] described hemorrhage *per diapedesis* in the rat subcutaneous tissue injected with the venom of *Bothrops jararaca*. An explanation for this apparent discrepancy was proposed by Gutiérrez *et al.* [49]. It was suggested that the predominant mechanism of extravasation depends on the type of microvessel being observed. In electron micrographs of capillary vessels, endothelial cell disruption occurs and *per rhexis* mechanism predominates. In contrast, in the studies of Ohsaka [13] and Gonçalves and Mariano [48], the affected microvessels shown in the micrographs in which erythrocytes escape through widened intercellular junctions are venules. Venules react to several inflammatory mediators by contraction and widening of intercellular junctions, leading to an increment in vascular permeability [50]. It has been described that erythrocyte extravasation may take place by the intercellular route in cases of intense inflammation [51], as occurs in snakebite envenoming. Thus, both mechanisms of extravasation, *i.e.*, *per rhexis* and *per diapedesis*, are likely to be involved in tissues as a consequence of the action of hemorrhagic SVMPs. In the case of skeletal muscle, ultrastructural observations strongly indicate that *per rhexis* mechanism predominates, and that the main locus of action of SVMPs is the capillary network, and not the venular part of the microvasculature.

Intravital microscopy has been a useful tool to investigate the actions of venom and toxins in tissues from a dynamic perspective. When the venom of *Bothrops asper* or isolated SVMPs from this venom have been applied to the mouse cremaster muscle, microhemorrhagic lesions were observed in capillary vessels few minutes after application [52,53]. These microbleedings occurred in an explosive fashion and resulted in burst-shaped microhematomas. As time passed, more microhematomas appeared in the capillary network. These observations tend to support the *per rhexis* mechanism of hemorrhage, since such microvascular hemorrhagic bursts are more compatible with a rupture in the integrity of the capillary wall than with a discrete extravasation of erythrocytes through widened intercellular junctions.

## 6. Are Endothelial Cells Directly Damaged by Hemorrhagic SVMPs? A Two-Step Hypothesis and the Role of Biophysical Factors Operating *in Vivo*

The described ultrastructural observations of microvessels affected by SVMPs, occurring within few minutes of injection, highlight a prominent early damage to endothelial cells *in vivo*. An obvious corollary of these observations is that hemorrhagic SVMPs are directly cytotoxic to endothelial cells. However, when SVMPs are incubated with endothelial cells in culture, no cytotoxicity is observed during the first hours of incubation, in contrast to the very rapid damage observed in these cells *in vivo*, which occurs within minutes [53,54,55,56]. The most evident consequence of the action of SVMPs on endothelial cells in culture is a detachment of these cells, as a consequence of cleavage of proteins in the substrate of culture wells [53,54,55]. Interestingly, detached cells remain viable several hours until, eventually, they undergo cell death by apoptosis. A number of SVMPs have been described to induce apoptosis in endothelial cells in culture [57,58,59,60]. It was proposed that apoptosis occurs by anoikis, *i.e.*, as a consequence of the detachment of cells from their substrate [59,60], although the observation of SVMP-induced cytotoxicity in non-adherent cells in suspension argues for other mechanisms of cell death in addition to anoikis [61]. The cellular mechanisms involved in SVMP-induced endothelial cell apoptosis have been explored in detail by several groups (see for example [58,59,60,61,62]).

The use of endothelial cell monolayers in culture as a model to investigate the action of SVMPs has the drawback that, phenotypically, these cells bear differences with microvascular endothelial cells *in vivo.* Baldo *et al.* [63] approached this limitation by using two-dimensional and three-dimensional cultures of endothelial cells in an extracellular matrix scaffold, this model being closer to the *in vivo* conditions of vascular endothelial cells. It was observed that collagen and Matrigel substrates enhanced endothelial cell damage induced by a hemorrhagic SVMP, allowing the binding of this enzyme to focal adhesions, disruption of stress fibers, detachment and apoptosis. This study stressed the relevance of developing *in vitro* models of endothelial cells which more closely resemble their phenotype *in vivo*. Nevertheless, even in these conditions, cytotoxic effects on endothelial cells appear after several hours and, therefore, do not reproduce the very early necrotic damage occurring in the microvasculature *in vivo* after injection of hemorrhagic SVMPs.

**Figure 2:**
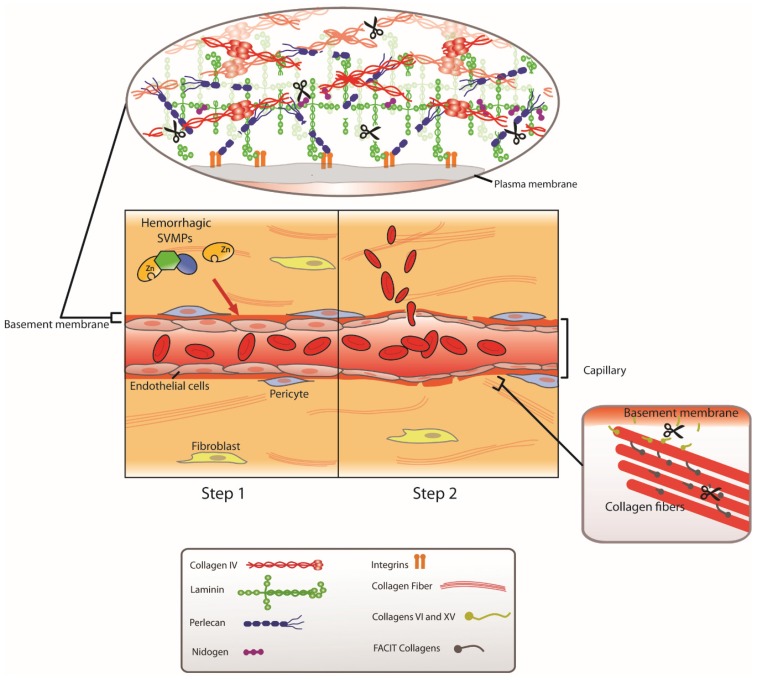
Two-step model to explain the mechanism of action of hemorrhagic SVMPs. The experimental evidence collected suggests that capillary vessel damage induced by hemorrhagic SVMPs occurs by a two-step mechanism. In the first step, SVMPs bind to and hydrolyze critical structural components of the basement membrane of capillary vessels, particularly type IV collagen and perlecan, and possibly other molecules that link the basement membrane to the fibrillar extracellular matrix. The cleavage of key peptide bonds of basement membrane components results in the mechanical weakening of this scaffold structure. As a consequence, in the second step, the biophysical hemodynamic forces normally operating in the microcirculation, *i.e.*, hydrostatic pressure, which largely determines wall tension, and shear stress, induce a distention of the vessel wall, until the capillary is eventually disrupted, with the consequent extravasation of blood. Reproduced by [68], Copyright 2011, Elsevier.

A hypothesis was proposed by Gutiérrez *et al.* [49] to explain this apparent discrepancy, by taking into consideration the possible role of the hemodynamic biophysical forces operating in the circulation *in vivo*, *i.e.*, wall tension and shear stress. According to Laplace’s law, wall tension is directly proportional to transmural pressure, *i.e.*, the difference between intravascular and extravascular hydrostatic pressures, and to the radius of the vessel [64]. In turn, shear stress is directly proportional to blood flow and viscosity, and inversely proportional to the vessel radius [65]. The distensibility of the vascular wall plays a key role in wall tension. In the case of capillaries, such distensibility is predominantly determined by the mechanical properties of their BM [66,67]. Thus, any effect in the mechanical stability of the BM would have a direct impact in the distensibility of the capillary wall. It was proposed by Gutiérrez *et al.* [49] that hemorrhagic SVMPs cause damage to the integrity of capillary vessels by a two-step mechanism: Initially, these enzymes hydrolyze key substrates at the BM surrounding endothelial cells in capillaries, causing a weakening in the mechanical stability of BM and increasing the distensibility of the microvessel wall. As a second step, the hemodynamic forces operating in the microcirculation, especially the hydrostatic force, induce a distention in the wall, which eventually culminates in the disruption of its integrity and in extravasation (Figure 2).

This hypothesis is compatible with ultrastructural alterations described above, *i.e.*, an increase in the capillary lumen associated with a thinning of endothelial cells and a drop in the number of pinocytotic vesicles, together with overt disruptions in the integrity of endothelial cells through which blood escapes to the interstitial space. One prediction arising from this hypothesis is that capillary damage should not occur in conditions of absence of blood flow. This was corroborated at the experimental level in muscle tissue of mice injected with a hemorrhagic SVMP in which blood flow was totally interrupted; no endothelial cell damage was observed in these conditions [47]. Thus, the rapid endothelial cell damage caused by hemorrhagic SVMPs *in vivo* is not due to a direct action of these enzymes in the cells, but instead to an indirect effect as a consequence of the weakening of the mechanical stability of the capillary wall owing to the effect of SVMP on the BM components. The obvious questions are, then: How do hemorrhagic SVMPs affect the BM? Why are some SVMP able to induce hemorrhage while others are not?

## 7. Exploring the Hydrolysis of Basement Membrane Components by SVMPs

The biochemical characterization of hemorrhagic components in snake venoms demonstrated that they are metalloproteinases. However, most of the early studies on purified SVMPs demonstrated proteolytic activity on a variety of substrates that do not correspond to the physiological targets of these enzymes [30,32]. Since microvessels are the target of hemorrhagic SVMPs, the study of degradation of BM components became a relevant task in understanding SVMPs’ mechanism of action. Ohsaka *et al.* [69] first demonstrated that hemorrhagic SVMPs hydrolyzed proteins of isolated glomerular BM. In 1988, Bjarnason *et al.* [70] described the ability of SVMPs to hydrolyze proteins of Matrigel, a BM preparation from Engelbreth–Holm–Swarm (EHS) mouse sarcoma, and on isolated BM components. Since then, several studies have described the hydrolysis of BM components by a number of SVMPs [53,71,72,73,74,75,76,77]. Notwithstanding, several puzzling findings arose from these observations: (a) The time-course of SVMP-induced hydrolysis of BM components *in vitro*, as judged by SDS-PAGE, usually take several hours, whereas hemorrhage *in vivo* appears within minutes; this could be explained on the grounds that SDS-PAGE analysis may not reveal subtle, but significant, early cleavage of BM components, which would be relevant for the weakening of BM stability. (b) Several SVMPs devoid or having very low hemorrhagic activity have been shown to hydrolyze BM components *in vitro* [78,79,80]. This raises the possibility that a critical attack to the BM mechanical stability might be related to the selective hydrolysis of some BM components.

The BM is a complex and highly specialized extracellular matrix structure which plays a key scaffold role in capillary endothelial cells and in other cell types [81]. BM contains major components such as laminin, type IV collagen, nidogen/entactin, and heparin sulphate proteoglycan (perlecan), in addition to a number of minor components such as agrin, APARC/BM-40/osteopontin, fibulins and types XV and XVIII collagens [81,82,83]. In the context of this complexity, which are the structurally relevant components whose cleavage by hemorrhagic SVMPs weakens the mechanical stability of microvessels? When comparing the hydrolysis of Matrigel *in vitro* by PI hemorrhagic and non-hemorrhagic SVMPs, a significant difference was found on the hydrolysis of type IV collagen and perlecan, since the former cleaved these substrates, particularly type IV collagen, to a greater extent [80]. On these grounds, deciphering the sites of cleavage of these molecules might shed light on the molecular basis of BM destabilization. Jay W. Fox, Jon Bjarnason and colleagues have studied the cleavage patterns of *Crotalus atrox* hemorrhagic SVMP on type IV collagen [32,72]. Cleavage occurs at the α1 (IV) and α2 (IV) chains. The α1 (IV) chains are cleaved at a triplet interruption region of the triple helix, whereas the α2 (IV) chain is cleaved in the triple helical region near the NC2 domain. Owing to the role of these sites in the association of type IV collagen monomers into tetramers, these cleavages may have implications on the mechanical stability of the BM [32].

## 8. Exploring the Hydrolysis of BM Components by SVMPs *in Vivo*

**Figure 3:**
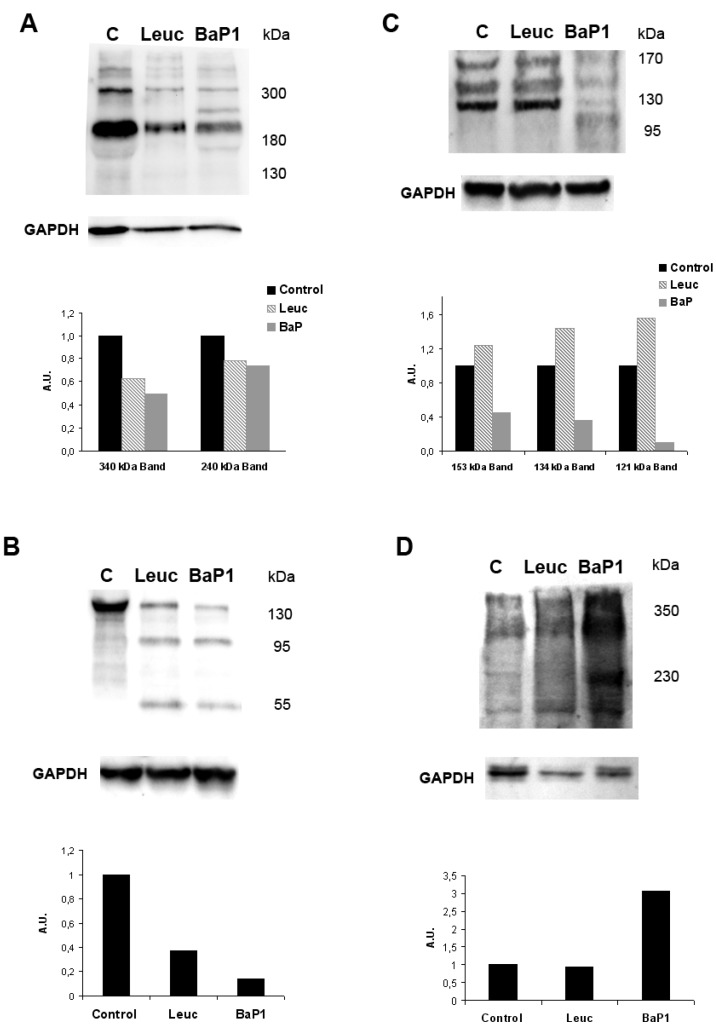
Differential hydrolysis of basement membrane components *in vivo* by hemorrhagic and non-hemorrhagic snake venom metalloproteinases (SVMPs). Non-hemorrhagic SVMP (leucurolysin a-leuc) and hemorrhagic SVMP (BaP1) were injected in the muscle of mice. After 15 min, animals were euthanized, and tissue was collected and homogenized. Supernatants of homogenates were separated by SDS-PAGE, and transferred to nitrocellulose membranes for immunodetection with either anti-laminin (**A**); anti-nidogen (**B**); anti-type IV collagen (**C**); and anti-endorepelin (perlecan) (**D**) antibodies, and with anti-GAPDH as loading control. (**C**) Control muscle injected with saline solution. A chemiluminiscent substrate was used to detect the reactions. Densitometric analysis was then performed. The molecular mass of various markers (in kDa) is shown at the right of the gels. A clear difference is observed between these SVMPs in the patterns of degradation of type IV collagen and endorepelin (perlecan). Reproduced by [80], Copyright 2011, PLOS.

The described *in vitro* observations of BM hydrolysis by SVMPs raised the need to explore the degradation of BM components *in vivo* as a consequence of injection of SVMPs. This has been approached by a combination of three methodologies, *i.e.*, immunohistochemistry in tissue sections, immunodetection by Western blot in tissue homogenates, and proteomics analysis of exudates collected in the vicinity of affected tissue. These complementary approaches have provided novel clues for understanding of the pathogenesis of SVMP-induced hemorrhage. Immunohistochemical staining of BM components as early as 15 min after injection of hemorrhagic SVMPs revealed loss of staining in microvessels of type IV collagen, laminin and nidogen [75,84], hence underscoring the rapid cleavage of these proteins. Such degradation was corroborated when performing immunoblots for detection of BM components on tissue homogenates and exudates [80,85]. Interestingly, when hemorrhagic and non-hemorrhagic SVMPs were compared, a similar pattern of hydrolysis was observed for laminin and nidogen, whereas the hemorrhagic enzyme induced a more extensive cleavage of perlecan and, especially, type IV collagen [80] (Figure 3).

Taken together, these findings strongly support the view that cleavage of type IV collagen, and probably perlecan as well, play a key role in the weakening of the mechanical stability of the BM of capillary vessels, and constitutes the first step in the pathogenesis of SVMP-induced microvessel damage [68,80]. When immunoblotting experiments were performed with muscle homogenates prepared from mice injected with a hemorrhagic SVMP in conditions where blood flow was interrupted, similar degradation patterns of BM components were observed (our unpublished results), but the integrity of capillary vessels was intact [47]. This supports the “two-step” hypothesis described above, since in conditions of no blood flow, the first step occurs, *i.e.*, enzymatic cleavage of BM components, but the second one, *i.e.*, distention and rupture of vessel integrity, does not, since the biophysical hemodynamic forces dependent on blood flow are not operating in these circumstances.

A significant step forward in the study of venom-induced tissue damage was the introduction of the proteomic analysis of exudate samples collected in the affected tissues [86], as well as of tissue homogenates [87]. This experimental approach allowed the identification of cleavage products of several extracellular matrix proteins, including BM components, as a consequence of the action of SVMPs [80,85,86]. In addition, fragments of type VI and type XV collagens were detected [80,85,86]. Interestingly, the amount of BM-specific heparan sulfate proteoglycan core protein, type VI collagen and type XV collagen were higher in exudates collected from tissue injected with a hemorrhagic SVMP than in samples injected with a non-hemorrhagic enzyme [80], thus suggesting that cleavage of these proteins may be related with the pathogenesis of hemorrhage. Type VI and XV collagens contribute to the stabilization of capillary vessels by connecting the BM with the surrounding extracellular matrix [88,89].

## 9. What Is the Basis for the Large Variation in the Hemorrhagic Potential of SVMPs?

When the hemorrhagic activity of isolated SVMPs is quantified, large differences are observed between enzymes. Moreover, some SVMPs are able to induce systemic hemorrhage, whereas others only cause local bleeding. The structural basis for these variations in hemorrhagic potential in enzymes that, otherwise, have similar cleavage patterns on protein substrates, has been deciphered as the structural features of SVMPs have become understood. In general, PIII SVMPs, which comprise Dis-like and Cys-rich domains in addition to the metalloproteinase domain, are more potent hemorrhagic toxins than PI SVMPs having only the catalytic domain [33,49]. Moreover, some of the few P-II SVMPs characterized to date are also potent hemorrhagic enzymes [90,91]. Thus, it is evident that the additional domains potentiate hemorrhagic activity, probably by the presence of relevant exosites in their sequences.

**Figure 4:**
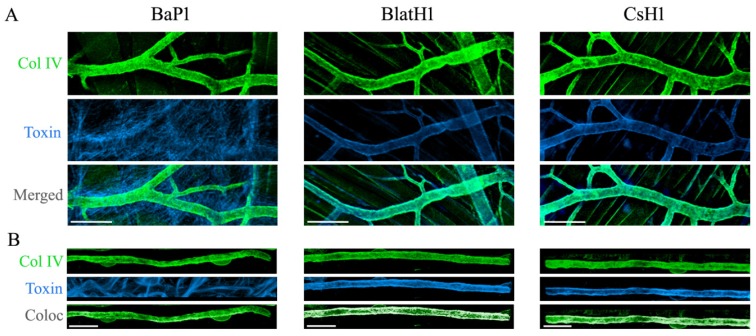
Immunolocalization of snake venom metalloproteinases (SVMPs) with vascular basement membrane on mouse cremaster muscle. Groups of mice were euthanized, and the cremaster muscle was dissected out. The isolated muscles were incubated for 15 min with equi-hemorrhagic amounts of three different SVMPs: BaP1 (PI SVMP, 30 µg), BlatH1 (PII SVMP, 3.5 µg) or CsH1 (PIII SVMP, 15 µg) labeled with Alexa Fluor^®^ 647 (blue). Control tissues were incubated with the SVMPs without labeling and no fluorescence was detected. Whole tissues were fixed with 4% paraformaldehyde and immunostained with anti-collagen IV following the secondary antibody labeled with Alexa Fluor 488 (green). Tissues were visualized in a Zeiss LSM 5 Pascal laser-scanning confocal microscope. Three-dimensional reconstitution of the images was carried out using IMARIS ×64 7.4.2 software. (**A**) Distribution of the SVMPs in the cremaster muscle tissue. Scale bar represents 150 µm. (**B**) Localization of SVMPs in capillary vessels in the cremaster. Scale bar represents 20 µm. White areas represent co-localization of the SVMPs (blue) with collagen IV (green) of vascular basement membrane in capillaries. Notice the predominant localization of PII and PIII SVMPs in the vasculature, whereas PI SVMP localizes in a more widespread fashion in the tissue. Reproduced by [85], Copyright 2015, PLOS.

These exosites may contribute to hemorrhagic activity in various ways. They may promote the binding of SVMPs to specific substrates in the tissues at relevant targets, such as the capillary microvascular network. Indeed, immunohistochemical evidence has demonstrated that PII and PIII SVMPs preferentially bind to the microvasculature when applied in mouse tissues, and present a pattern of co-localization with type IV collagen in the vessel wall [84,85] (Figure 4). The same pattern of co-localization was observed for a fragment constituted by the Dis-like and Cys-rich domains (DC fragment) of a PIII SVMP, confirming that sequences in these domains are responsible for the targeting to microvessels [84]. In contrast, PI SVMPs, devoid of these additional domains, show a widespread localization in the extracellular matrix and are not concentrated in the vessels [84,85].

Biochemical studies with jararhagin, a hemorrhagic PIII SVMPs from the venom of *Bothrops jararaca*, showed that the Dis-like and Cys-rich domains mediate the binding to collagens and α_2_β_1_ integrin, respectively [92,93]. Moreover, the Cys-rich domain of PIII SVMPs is responsible for the binding to type I collagen and several proteins presenting von Willebrand factor (vWF) A domains, such as vWF, various fibrillary-associated collagens with interrupted triple helices (FACITs) and matrylins [94,95,96]. In light of the pathological and proteomic observations described above, the binding to various fibrillar and non-fibrillar collagens are relevant, since hydrolysis of these substrates is likely to be causally related to the onset of microvessel damage [68]. The fact that PII SVMPs are also potent hemorrhagic toxins, and at least one of them co-localizes with type IV collagen in the vessels [85], demonstrates that exosites located in the Dis domain allow the targeting of these SVMPs to the microvasculature. Thus, by directing PII and PIII SVMPs to the microvessels, these additional domains are likely to position the catalytic sites of these enzymes nearby relevant substrates in the BM. In contrast, PI SVMPs are not targeted to these loci in the tissue and therefore are less effective in causing capillary damage.

It has been also described that the generalist plasma proteinase inhibitor α_2_-macroglobulin (α_2_M) effectively abrogates proteolytic and hemorrhagic activities of PI SVMPs, whereas it is ineffective to inhibit these activities in PII and PIII SVMPs [91,97,98,99,100]. The structural basis for this difference remains unknown, but it is likely that structural constraints in the Dis, Dis-like and Cys-rich domains impair the inhibitory action of α_2_M. This finding is relevant in the light of the ability of PII and PIII SVMPs to induce systemic hemorrhage, since they are not inhibited upon their entrance into the circulation and their distribution to various organs. In contrast, PI SVMPs are readily inhibited by α_2_M when they reach the bloodstream, thereby precluding their action at distant organs [49]. Moreover, PII and PIII SVMPs are able to inhibit platelet aggregation, owing to the action of sequences of their Dis, Dis-like and Cys-rich domains [101], and also have the ability to degrade vWF [96,102]. However, these effects on hemostasis have been shown *in vitro*, and it remains to be investigated whether they also have an impact *in vivo*.

## 10. SVMP-Induced Hemorrhage Viewed from a Holistic Perspective: Studying the Action of SVMP in the Context of the Whole Venom

The field of Toxinology, as many other areas in biomedical research, is moving from a predominantly “reductionist” approach to a more “holistic” and integrated perspective [103]. In this context, the characterization of the structural and functional properties of hemorrhagic SVMPs has provided a rich body of knowledge that has expanded our understanding on the mechanism of action of these toxins in the tissues and on the structural determinants of their activity. Nevertheless, in actual snakebites, it is whole venom that is injected in the victim’s tissues and, consequently, the action of toxins has to be analyzed in the context of venoms. Probably the best example of this need, in the case of hemorrhagic activity of SVMPs, is the potentiation of this activity by venom components that affect hemostasis. Viperid venoms contain an array of proteins acting on plasma coagulation factors and platelets [2,101,104,105]. Serine proteinases and SVMPs exert procoagulant effects by displaying thrombin-like activity, or by activating factors II, X or V of the coagulation cascade [106]. *In vivo*, these procoagulant effects result in defibrinogenation and incoagulability [2,105]. Although incoagulability *per se* does not necessarily result in significant bleeding, coagulopathy potentiates bleeding in the context of SVMP-induced vascular damage. In some cases, the action of procoagulant components in snake venoms or the damage to the endothelial lining cause regional thrombosis [107,108], with the consequent local or remote ischemic events, which contribute to the complexity of the pathophysiology of cardiovascular alterations in snakebite envenomings.

Likewise, various venom components, such as proteins of the C-type lectin-like family, and also serine proteinases, SVMPs, and disintegrins, exert effects that impair platelet function, either by causing thrombocytopenia or inhibition of platelet aggregation [101,109,110,111,112]. The role of venom-induced platelet disturbances in potentiating hemorrhagic activity has been demonstrated clinically [113] and at the experimental level, whereby mice rendered thrombocytopenic by a C-type lectin-like venom component developed stronger hemorrhage in the lungs after injection with a hemorrhagic SVMP [114].

Less evident instances of synergisms between SVMPs and other venom components have been also described. The co-administration of hyaluronidase and a hemorrhagic SVMP increased the hemorrhagic area as compared to the action of the SVMP alone, thus suggesting that hyaluronidase plays the role of a spreading factor that extends the action of SVMPs [115,116]. In this context, it is relevant to consider the possible involvement of non-hemorrhagic SVMPs since these enzymes, despite lacking hemorrhagic activity, degrade various extracellular matrix components [79]; this effect might contribute to the spreading and action of hemorrhagic SVMPs, a hypothesis that requires experimental analysis. More recently, a puzzling phenomenon was described whereby a non-toxic venom phospholipase A_2_, which does not affect endothelial cells, potentiates the cell-detaching effect induced by a hemorrhagic SVMP on an endothelial cell line in culture [117]. Moreover, the inflammatory landscape generated by various components upon venom injection in the tissues is likely to promote vascular alterations that might potentiate the disruptive effect of hemorrhagic SVMPs in the microvasculature. The role of inflammation in the pathogenesis of systemic hemorrhage in organs such as the lungs or the brain remains to be explored.

## 11. Concluding Remarks

This summarized account of the journey followed by toxinologists for understanding the mechanisms by which SVMPs induce hemorrhage has provided an overview of some of the landmarks in the characterization of SVMPs and their action in the microvasculature. It is now clear that hemorrhage occurs mostly through the SVMP-induced degradation of key structural proteins at the BM and its surroundings. The mechanical weakening generated by this enzymatic degradation results in microvessel wall distention owing to the action of the hemodynamic forces operating in the circulation, with the eventual disruption of the capillary wall and extravasation. The wide variation in the hemorrhagic potential of SVMPs of different domain composition has been explained by the role of Dis, Dis-like and Cys-rich domains in positioning these enzymes at physiologically-relevant sites for exerting their hemorrhagic activity, *i.e.*, at the microvasculature. There is a need to explore, at deeper levels, which are the BM components critical for the disruption of microvessel integrity; in this regard, the use of immunoelectron microscopic techniques should provide novel information on the precise localization of SVMPs in the BM. Likewise, the proteomic analysis of samples of exudate and tissue collected after injection of SVMPs is likely to offer further details of the fine pathological alterations induced by these enzymes. The use of diverse, and complementary, methodological platforms has been helpful in generating valuable information on the mechanism of action of SVMPs, and should be strengthened and expanded in the future.

On the other hand, the action of hemorrhagic SVMPs in the organism has to be studied from a more holistic perspective, by understanding it in the overall context of envenoming. In this regard, the study of pathological alterations caused by SVMPs has to be complemented with the study of these effects induced by crude venoms and by combinations of various venom components, as well as by understanding the role of tissue responses to envenoming in the pathogenesis of hemorrhage and other pathological effects. The complexity of these tasks demands the integration of multidisciplinary research tools, including informatic resources. Toxinology is in need of renewed conceptual and experimental platforms aimed at reaching a more profound understanding of the highly complex pathophysiology of snakebite envenoming, including the pathogenesis of hemorrhage.
